# Major Andre’s Remains

**Published:** 1881-07

**Authors:** 


### MAJOR ANDRE'S REMAINS.

Twenty-four miles up the Hudson from New York is the ancient town of Tappan, lying about four miles back from the river. This place, it will be remembered, was the headquarters of General Washington at the time of Arnold's treason, and the scene of the execution of the gifted but unfortunate young Major Andre.

The spot of Andre's execution was upon an elevation where it could be witnessed by the whole army, who were encamped around in the vicinity.

His remains were removed to England in 1831, and placed near his monument in Westminister Abbey.

It was remarkable that any considerable portion of the remains should have been found after a lapse of so long a period.

The grave was located in the centre of a cultivated field of about a dozen acres, but the plowshare had never been permitted to approach within four yards of the spot.

After digging to the depth of three or four feet, the coffin was reached, and, on being opened, the entire skeleton was discovered, all the bones in the most perfect order, and the root of a peach tree, having protruded through the coffin, forming a network around the skull. He was found to have been a man of small stature

There had been a tradition that Andre's remains were removed to England many years before, which was repeated at the time ; but, on investigation into the matter, it was put to rest by some of the aged inhabitants, who were present at the interment, and who were satisfied the grave had never been reopened.

After removing the remains to the sarcophagus provided for their reception, search was made among the dust in the coffin to ascertain if he was buried in his uniform ; but no buttons were found, and it was afterward remembered by an aged resident that his uniform had been given to his servant.

